# Supplementary material for: Do family physicians with focused practice or Care of the Elderly training practice differently than others? A population-based, propensity score-matched cohort study
Source: PLoS One. 2026 May 27;21(5):e0347828. doi: 10.1371/journal.pone.0347828 (PMC13215490; doi:10.1371/journal.pone.0347828)
Supplement: S1 File — Contains the completed RECORD checklist (S1 Appendix), description of relevant datasets (S2 Appendix), technical definitions of process measures (S3 Appendix), summary of missing data (S4 Appendix), and sensitivity analysis (S5 Appendix). (DOCX) [file pone.0347828.s001.docx]

**S1 File. Supporting Information File**

**S1 Appendix. Completed RECORD checklist**

|  | **Item No.** | **STROBE items** | **Location in manuscript where items are reported** | **RECORD items** | **Location in manuscript where items are reported** |
| --- | --- | --- | --- | --- | --- |
| **Title and abstract** | | | | | |
|  | 1 | (a) Indicate the study’s design with a commonly used term in the title or the abstract (b) Provide in the abstract an informative and balanced summary of what was done and what was found | Page 1-2 | RECORD 1.1: The type of data used should be specified in the title or abstract. When possible, the name of the databases used should be included.  RECORD 1.2: If applicable, the geographic region and timeframe within which the study took place should be reported in the title or abstract.  RECORD 1.3: If linkage between databases was conducted for the study, this should be clearly stated in the title or abstract. | Page 2  Page 2  Page 2 |
| **Introduction** | | | | | |
| Background rationale | 2 | Explain the scientific background and rationale for the  investigation being reported | Pages 3-4 |  |  |
| Objectives | 3 | State specific objectives, including any prespecified hypotheses | Page 4 |  |  |
| **Methods** | | | | | |
| Study Design | 4 | Present key elements of study design early in the paper | Page 4 |  |  |
| Setting | 5 | Describe the setting, locations, and relevant dates, including  periods of recruitment, exposure, follow-up, and data collection | Page 4 |  |  |

| Participants | 6 | 1. *Cohort study* - Give the eligibility criteria, and the sources and methods of selection of participants. Describe methods of follow-up 2. *Cohort study* - For matched studies, give matching criteria and number of exposed and unexposed | Pages 4-5  Page 6, Figure 1 | RECORD 6.1: The methods of study population selection (such as codes or algorithms used to identify subjects) should be listed in detail. If this is not possible, an explanation should be provided.  RECORD 6.2: Any validation studies of the codes or algorithms used to select the population should be referenced. If validation was conducted for this study and not published elsewhere, detailed methods and results should be provided.  RECORD 6.3: If the study involved linkage of databases, consider use of a flow diagram or other graphical display to demonstrate the data linkage process, including the number of individuals with linked data at each stage. | Pages 4-5, S2 Appendix  Pages 4-5  Page 4, Figure 1 |
| --- | --- | --- | --- | --- | --- |
| Variables | 7 | Clearly define all outcomes, exposures, predictors, potential confounders, and effect modifiers. Give diagnostic criteria, if applicable. | S2 and S3 Appendix | RECORD 7.1: A complete list of codes and algorithms used to classify exposures, outcomes, confounders, and effect modifiers should be provided. If these cannot be reported, an explanation should be provided. | S2 and S3 Appendix |
| Data sources/ measurement | 8 | For each variable of interest, give sources of data and details of methods of assessment (measurement).  Describe comparability of assessment methods if there is more than one group | Page 4, S2 and S3 Appendix |  |  |
| Bias | 9 | Describe any efforts to address potential sources of bias | Page 6 |  |  |
| Study size | 10 | Explain how the study size was arrived at | Pages 4-5 |  |  |

| Quantitative variables | 11 | Explain how quantitative variables were handled in the analyses. If applicable, describe  which groupings were chosen, and why | Pages 4-6 |  |  |
| --- | --- | --- | --- | --- | --- |
| Statistical methods | 12 | 1. Describe all statistical methods, including those used to control for confounding 2. Describe any methods used to examine subgroups and interactions 3. Explain how missing data were addressed 4. *Cohort study* - If applicable, explain how loss to follow-up was addressed 5. Describe any sensitivity analyses | Pages 6-7 |  |  |
| Data access and cleaning methods |  | .. |  | RECORD 12.1: Authors should describe the extent to which the investigators had access to the database population used to create the study population.  RECORD 12.2: Authors should provide information on the data cleaning methods used in the study. | Pages 4-5  Pages 4-6 |
| Linkage |  | .. |  | RECORD 12.3: State whether the study included person-level, institutional-level, or other data linkage across two or more databases. The methods of linkage and methods of linkage quality evaluation should be provided. | Page 4 |

|  | | **Results** | | | | | |  |
| --- | --- | --- | --- | --- | --- | --- | --- | --- |
| Participants | | | 13 | 1. Report the numbers of individuals at each stage of the study (*e.g.*, numbers potentially eligible, examined for eligibility, confirmed eligible, included in the study, completing follow-up, and analysed) 2. Give reasons for non- participation at each stage. 3. Consider use of a flow diagram | Figure 1 , Page 7 | RECORD 13.1: Describe in detail the selection of the persons included in the study (*i.e.,* study population selection) including filtering based on data quality, data availability and linkage. The selection of included persons can be described in the text and/or by means of the study flow diagram. | Figure 1 |  |
| Descriptive data | | | 14 | 1. Give characteristics of study participants (*e.g.*, demographic, clinical, social) and information on exposures and potential confounders 2. Indicate the number of participants with missing data for each variable of interest 3. *Cohort study* - summarise follow-up time (*e.g.*, average and total amount) | Page 7, Table 1  Page 7  N/A |  |  |  |
| Outcome data | | | 15 | *Cohort study* - Report numbers of outcome events or summary measures over time | Pages 8-9, Table 2 |  |  |  |
| Main results | | | 16 | 1. Give unadjusted estimates and, if applicable, confounder- adjusted estimates and their precision (e.g., 95% confidence interval). Make clear which confounders were adjusted for and why they were included 2. Report category boundaries when continuous variables were categorized | Page 9, Table 2 |  |  |  |
| Other analyses | | | 17 | Report other analyses done— e.g., analyses of subgroups and interactions, and sensitivity analyses | N/A |  |  |  |
|  | | **Discussion** | | | | | |  |
| Key results | | | 18 | Summarise key results with  reference to study objectives | Page 12 |  |  |  |
| Limitations | | | 19 | Discuss limitations of the study, taking into account sources of potential bias or imprecision.  Discuss both direction and magnitude of any potential bias | Page 15 | RECORD 19.1: Discuss the implications of using data that were not created or collected to answer the specific research question(s). Include discussion of misclassification bias, unmeasured confounding, missing data, and changing eligibility over time, as they pertain to the study being  reported. | Page 15 |  |
| Interpretation | | | 20 | Give a cautious overall interpretation of results considering objectives, limitations, multiplicity of analyses, results from similar studies, and other relevant evidence | Pages 12-14 |  |  |  |
| Generalisability | | | 21 | Discuss the generalisability (external validity) of the study results | Pages 14-15 |  |  |  |
|  | **Other Information** | | | | | | | |
| Funding | | | 22 | Give the source of funding and the role of the funders for the present study and, if applicable, for the original study on which the present article is based | Submission platform |  |  |  |
| Accessibility of protocol, raw data, and programming  code | | |  | .. |  | RECORD 22.1: Authors should provide information on how to access any supplemental information such as the study protocol, raw data, or  programming code. | Supplementary Information file |  |

**S2 Appendix. Description of relevant datasets**

| **Data sets** | **Description** | **Relevant variables** |
| --- | --- | --- |
|  |  |  |
| Ontario Health Insurance Plan (OHIP) | The OHIP claims database contains information on publicly funded health services, primarily provided by physicians, to Ontario residents eligible for provincial health insurance. The main data elements are service/billing codes submitted by physicians. | - **Cohort creation:** physicians who submitted at least one OHIP fee claim in 2019 with “00” specialty code - **Cohort classification:** focused practice in COE fee codes (i.e., A967, K703, K077, B988, B986) - **Baseline characteristics:** long-term care practice^[[1]](#footnote-1)^ - **Outcomes:** physician fee codes |
| College of Family Physicians of Canada (CFPC) Membership Database | The CFPC Membership Database is based on an annual survey to clinical and research members. We imported and linked a subset of membership data pertaining to COE CAC holders. | - **Cohort classification:** validated list of COE CAC holders |
| ICES Physician Database (IPDB) | IPDB uses billing and workforce information to assign specialties based on the proportion of specialty billing codes used. | - **Baseline characteristics:** years in clinical practice, community size of primary practice location, practice type, affiliation with a patient enrolment model |
| Primary Care Population (PCPOP) | PCPOP is an ICES-derived cohort of Ontario residents who are eligible for provincial health insurance and had at least one health services encounter within nine years of the index event. | - **Baseline characteristics:** number of patients aged ≥65 |
| Client Agency Program Enrolment (CAPE) | CAPE lists patients registered with a primary care organization and contains information on patients’ association to a specific physician and primary care organization. | - **Outcomes:** number of attached patients |
| Ontario Drug Benefit (ODB) | ODB contains information (recipients, payments, claims, practitioners) for the Ontario Drug Benefit Program. Drug identification number is provided by IQVIA Solutions Canada Inc. | - **Outcomes:** drug identification numbers |
| ICES-derived cohorts | Validated cohorts of individuals with specific diseases and conditions, including the Congestive Heart Failure (CHF) database, Chronic Obstructive Pulmonary Disease (COPD) database, and Ontario Dementia Dataset (DEMENTIA). | - **Outcomes:** patients diagnosed with dementia, congestive heart failure, and chronic obstructive pulmonary disease |
| Discharge Abstract Database (DAD) | The DAD contains patient-level clinical, demographic, diagnostic, procedural, and treatment data for hospital admissions and day surgeries. | - **Outcomes:** potentially inappropriate medication use |
| Same Day Surgery (SDS) | SDS contains patient-level demographic, diagnostic, procedural and treatment information on all day surgeries. | - **Outcomes:** potentially inappropriate medication use |
| National Ambulatory Care Reporting System (NACRS) | NACRS contains patient-level demographic, diagnostic, procedural and treatment information for all hospital-based and community-based ambulatory care, including outpatient and community-based clinics and emergency departments. | - **Outcomes:** potentially inappropriate medication use |

COE=Care of the Elderly; CAC=Certificate of Added Competence

**S3 Appendix. Technical definitions of process measures**

| **Indicator #** | **Technical definition** | | | |
| --- | --- | --- | --- | --- |
| 1 | Indicator name | Older adults who receive the influenza immunization. | | |
|  | Description | The proportion of attached older adults to a family physician with a billing record for influenza immunization. | | |
|  | Calculation | Proportion, % (Numerator divided by denominator x 100%) | | |
|  | Numerator | The number of older adults (aged ≥65) with a billing record for influenza immunization in 2019. | | |
|  |  | Data set(s) | OHIP fee codes: | ODB DINs: |
|  |  | Variable(s) | - G590 (Immunization of influenza agent), - G592 (Administration of intranasal influenza vaccine), - Q130 (Preventative care tracking code), **or** - G538 (Other immunization) | - 02015986, 02223929, 02269562, 02346850, 02362384, 02365936, 02420643, 02420686, 02420783, 02426544, 02428881, 02432730, 02445646, 02473283, 02473313, 02494248, 02500523, 09857501, 09857645, 09857646, **or** 09858149 |
|  | Denominator | The number of older adults (aged ≥65) attached (formally rostered or virtually) to a particular family physician in 2019. | | |
|  |  | Data set(s) | CAPE (main source for rostering information)  OHIP (virtual rostering information) | |
|  | Considerations and limitations | 1. This indicator only reflects influenza immunizations associated with a physician fee code (i.e., billing record) or DIN for pharmacist-administered vaccinations. Therefore, it excludes vaccines administered in workplaces, hospitals, or public health by other health professionals (e.g., registered nurses) where the family physician does not subsequently bill. 2. While G538 in OHIP captures immunizations where the type is not stated, it is not specific to influenza immunizations. However, these differences in specificity are likely balanced between groups. 3. In some team-based models (e.g., FHTs), physicians may not bill for immunizations administered by others in the practice (e.g., nurse practitioners). | | |
|  | References | Canadian Institute for Health Information. (2016). Pan-Canadian Primary Health Care Indicator Update Report. Ottawa, Canada. Available: <https://secure.cihi.ca/free_products/Pan-Canadian_PHC_Indicator_Update_Report_en_web.pdf> | | |
| 2 | Indicator name | Older adults living with COPD who receive influenza and pneumococcal immunizations. | | |
|  | Description | The proportion of older adults living with COPD who receive influenza and pneumococcal immunizations in 2019. | | |
|  | Calculation | Proportion, % (Numerator divided by denominator x 100%) | | |
|  | Numerator | The number of older adults (aged ≥65) whose family physician billed for administering influenza and pneumococcal immunizations in 2019. | | |
|  |  | Data set(s) | OHIP fee codes: | ODB DINs: |
|  |  | Variable(s) | - G846 (Pneumococcal Conjugate), - G590 (Immunization of influenza agent), - G592 (Administration of intranasal influenza vaccine), **or** - Q130 (Preventative care tracking code) | - 02015986, 02223929, 02269562, 02346850, 02362384, 02365936, 02420643, 02420686, 02420783, 02426544, 02428881, 02432730, 02445646, 02473283, 02473313, 02494248, 02500523, 09857501, 09857645, 09857646, **or** 09858149 |
|  |  |  | Note: OHIP/ODB SERVDATE for activities occurred after dementia diagnosis date. | |
|  | Denominator | The number of older adults (aged ≥65) living with COPD attached (formally rostered or virtually) to a particular family physician in 2019. | | |
|  |  | Data set(s) | CAPE (main source for rostering information)  OHIP (virtual rostering information)  COPD (ICES-derived cohort) | |
|  | Considerations and limitations | 1. This indicator only reflects influenza immunizations associated with a physician fee code (i.e., billing record) or DIN for pharmacist-administered vaccinations. Therefore, it excludes vaccines administered in workplaces, hospitals, or public health by other health professionals (e.g., registered nurses) where the family physician does not subsequently bill. 2. While G538 in OHIP captures immunizations where the type is not stated, it is not specific to influenza immunizations. However, these differences in specificity are likely balanced between groups. 3. In some team-based models (e.g., FHTs), physicians may not bill for immunizations administered by others in the practice (e.g., nurse practitioners). | | |
|  | References | Canadian Institute for Health Information. (2016). Pan-Canadian Primary Health Care Indicator Update Report. Ottawa, Canada. Available: <https://secure.cihi.ca/free_products/Pan-Canadian_PHC_Indicator_Update_Report_en_web.pdf> | | |
| 3 | Indicator name | Older adults living with dementia who receive tests aligned with the most current Canadian Consensus on Dementia. | | |
|  | Description | The proportion of older adults living with dementia who receive tests aligned with the most current Canadian Consensus on Dementia in 2019. | | |
|  | Calculation | Proportion, % (Numerator divided by denominator x 100%) | | |
|  | Numerator | The number of older adults (aged ≥65) whose family physician billed for tests aligned with the most current Canadian Consensus on Dementia. | | |
|  |  | Data set(s) | OHIP fee codes: | |
|  |  | Variable(s) | - K032 (Extended specific neurocognitive assessment), - X421 (Head MRI multi-slice sequence), **or** - X425 (Head MRI repeat) | |
|  | Denominator | The number of older adults (aged ≥65) living with dementia attached (formally rostered or virtually) to a particular family physician in 2019. | | |
|  |  | Data set(s) | CAPE (main source for rostering information)  OHIP (virtual rostering information)  DEMENTIA (ICES-derived cohort) | |
|  | Considerations and limitations | 1. Many of the recommendations from the most current Canadian Consensus on Dementia cannot be measured using administrative data (e.g., patients’ symptoms warranting cognitive testing). 2. The family physician may not have ordered the test; the indicator may not accurately reflect their clinical practice (e.g., ordered by neurologist). | | |
|  | References | Rojas-Rozo, L., Lee, L., Khanassov, V., Sivananthan, S., Ismail, Z., Gauthier, S., & Vedel, I. (2023). Latest Canadian Consensus Conference on the Diagnosis and Treatment of Dementia: What’s in It for Primary Care?. Canadian Journal on Aging, 19: 1-12. 10.1017/S0714980823000521 | | |
| 4 | Indicator name | Older adults living with dementia who receive dementia care management. | | |
|  | Description | The proportion of older adults living with dementia who are attached to a family physician who provided dementia care management in 2019. | | |
|  | Calculation | Proportion, % (Numerator divided by denominator x 100%) | | |
|  | Numerator | The number of older adults (aged ≥65) whose family physician billed for activities related to dementia care in 2019. | | |
|  |  | Data set(s) | OHIP fee codes: | |
|  |  | Variable(s) | - K035 (Mandatory reporting of medical condition to the Ontario), - A900 (Complex house call assessment), - K032 (Extended specific neurocognitive assessment), - K033 (Individual counselling), - K013 (Individual counselling), - K703 (Geriatric outpatient case conference), - K132 (General assessment for an adult 65 years of age and older), - A967 (Care of the Elderly Focused Practice Assessment), - A003 (General assessments), - A004 (General re-assessments), - A007 (Intermediate assessments), - K077 (Geriatric telephone support), - K005 (Primary mental health care visit for dementia), **or** - K070 (home care application)   Note: OHIP SERVDATE for activities occurred after dementia diagnosis date. | |
|  | Denominator | The number of older adults (aged ≥65) living with dementia attached (formally rostered or virtually) to a particular family physician in 2019. | | |
|  |  | Data set(s) | CAPE (main source for rostering information)  OHIP (virtual rostering information)  DEMENTIA (ICES-derived cohort) | |
|  | Considerations and limitations | 1. There are some concerns about construct validity as the ICES-derived cohort for dementia patients is derived from some physician fee codes for activities that would constitute “dementia care management.” 2. While some of the fee codes are not specific to “dementia care management” activities, they all pertain to activities in the care of persons living with dementia. | | |
|  | References | Godard-Sebillotte, C., Le Berre, M., Schuster, T., Trottier, M., & Vedel, I. (2019). Impact of health service interventions on acute hospital use in community-dwelling persons with dementia: A systematic literature review and meta-analysis. PLoS One, 14(6), e0218426. | | |
| 5 | Indicator name | Older adults who are prescribed benzodiazepines. | | |
|  | Description | The proportion of attached older adults to a family physician who have been prescribed one or more benzodiazepines in 2019. | | |
|  | Calculation | Proportion, % (Numerator divided by denominator x 100%) | | |
|  | Numerator | The number of older adults (aged ≥65) with a prescription for one or more benzodiazepines in 2019. | | |
|  |  | Data set(s) | ODB DINs: | |
|  |  | Variable(s) | - 00548359, 00548367, 00677477, 00677485, 00813958, 00865397, 00865400, 01913239, 01913247, 01913484, 01913492, 02137534, 02137542, 02230074, 02230075, 02349191, 02349205, 02400111, 02400138, 02417634, 02417642, 00518123, 00518131, 00682314, 02167808, 02167816, 02167824, 02171856, 02171864, 02171872, 02177153, 02177161, 02177188, 02192705, 02192713, 02192721, 02230584, 02230585, 00522724, 00522988, 00522996, 00012645, 00295051, 00012629, 00012637, 00013463, 00013471, 00013498, 00020915, 00020923, 00020931, 00398403, 00398411, 00398438, 00115630, 01989634, 02221799, 02238334, 02238797, 02244474, 02244638, 00846392, 00382825, 00382841, 02048701, 02048736, 02103656, 02103737, 02145227, 02145243, 02173344, 02173352, 02177889, 02177897, 02207818, 02230366, 02230369, 02230950, 02230951, 02233960, 02233985, 02236948, 02239024, 02239025, 02270641, 02270676, 02303337, 02345676, 09852395, 00264911, 00264938, 00264946, 00628190, 00628204, 00628212, 00860689, 00860697, 00860700, 02386143, 00012874, 00399728, 00602825, 02065614, 09857240, 00891797, 02238162, 09853340, 09853430, 00013277, 00013285, 00013293, 00013757, 00013765, 00013773, 00272434, 00272442, 00272450, 00272639, 00272647, 00280429, 00362158, 00396230, 00405329, 00405337, 00466891, 00466905, 00012696, 00012718, 00496545, 00496553, 00521698, 00521701, 00483818, 00483826, 00514519, 00514527, 00557773, 02041405, 02243278, 09857216, 02410753, 02410761, 00348325, 00348333, 00399124, 00637742, 00637750, 00655740, 00655759, 00655767, 00711101, 00728187, 00728195, 00728209, 00865672, 00865680, 00865699, 02041413, 02041421, 02041448, 02351080, 02351099, 00557757, 00557765, 00722138, 02041456, 02041464, 02041472, 02240285, 02240286, 02243254, 09857225, 00766011, 00784516, 02242905, 09857436, 09857437, 09857438, 00511528, 00511536, 02229654, 02229655, 02234003, 02234007, 02245230, 02245231, 00231363, 00295698, 00295701, 00402680, 00402737, 00402745, 00483893, 00483907, 00483915, 00496529, 00496537, 00500852, 02043653, 02043661, 02043688, 00604453, 00604461, 02223570, 02223589, 02225964, 02225972, 02229455, 02229456, 02230095, 02230102, 02231615, 02231616, 02243023, 02243024, 02244814, 02244815, 02273039, 02273047, 00443158, 00512559, 00614351, 00614378, 00808563, 00808571, 00872431, 00886084, 00886092, 01913506, 01995227, 02230024, **or** 02230025 | |
|  | Denominator | The number of older adults (aged ≥65) attached (formally rostered or virtually) to a particular family physician in 2019. | | |
|  |  | Data set(s) | CAPE (main source for rostering information)  OHIP (virtual rostering information) | |
|  | Considerations and limitations | 1. Without knowledge of patients’ clinical or behavioural symptoms, we do not know whether instances of prescribing benzodiazepines were warranted and appropriate (e.g., for insomnia, agitation, seizure disorder, alcohol withdrawal). 2. The family physician may not have prescribed the benzodiazepine; the indicator may not accurately reflect their clinical practice. | | |
|  | References | This DIN list was based on an extensive search in the ODB at ICES (using the %dinexplore macro) for all active and relevant drugs, and reviewed for accuracy by a physician. | | |
| 6 | Indicator name | Older adults who are prescribed medications with strong anticholinergic effects. | | |
|  | Description | The proportion of attached older adults to a family physician who have been prescribed one or more medications with strong anticholinergic effects in 2019. | | |
|  | Calculation | Proportion, % (Numerator divided by denominator x 100%) | | |
|  | Numerator | The number of older adults (aged ≥65) with a prescription for one or more medications with strong anticholinergic effects in 2019. | | |
|  |  | Data set(s) | ODB DINs: | |
|  |  | Variable(s) | - 00706531, 00649392, 01927744, 00545058, **or** 00545074 | |
|  | Denominator | The number of older adults (aged ≥65) attached (formally rostered or virtually) to a particular family physician in 2019. | | |
|  |  | Data set(s) | CAPE (main source for rostering information)  OHIP (virtual rostering information) | |
|  | Considerations and limitations | 1. Without knowledge of patients’ clinical or behavioural symptoms, we do not know whether instances of prescribing anticholinergics were warranted and appropriate (e.g., for cognitively intact person with an overactive bladder). 2. Unable to capture instances where these medications are obtained over the counter (e.g., Gravol, Benadryl); although this does not reflect the family physician’s clinical practice. 3. The family physician may not have prescribed the anticholinergic; the indicator may not accurately reflect their clinical practice. | | |
|  | References | This DIN list was based on an extensive search in the ODB at ICES (using the %dinexplore macro) for all active and relevant drugs, and reviewed for accuracy by a physician. | | |
| 7 | Indicator name | Older adults who are prescribed potentially inappropriate medications. | | |
|  | Description | The proportion of attached older adults to a family physician who have been prescribed one or more potentially inappropriate medications in 2019. | | |
|  | Calculation | Proportion, % (Numerator divided by denominator x 100%) | | |
|  | Numerator | The number of older adults (aged ≥65) with a prescription for one or more potentially inappropriate medications in 2019. | | |
|  |  | Data set(s) | ODB, DAD, SDS, NACRS, OHIP | |
|  |  | Variable(s) | We utilized the DINs specified by Bjerre et al. (below) to identify potentially inappropriate medications. | |
|  | Denominator | The number of older adults (aged ≥65) attached (formally rostered or virtually) to a particular family physician in 2019. | | |
|  |  | Data set(s) | CAPE (main source for rostering information)  OHIP (virtual rostering information) | |
|  | Considerations and limitations | 1. Without knowledge of patients’ clinical or behavioural symptoms, we do not know whether instances of prescribing potentially inappropriate medications were warranted and appropriate (e.g., for urinary incontinence). 2. Since there are many lists of potentially inappropriate medications (e.g., Beers, STOPP/START, medication appropriateness index), we selected to utilize the medications specified by Bjerre et al. 3. The family physician may not have prescribed the potentially inappropriate medication; the indicator may not accurately reflect their clinical practice. | | |
|  | References | 1. Bjerre, L.M., Ramsay, T., Cahir, C., Ryan, C., Halil, R., Farrell, B., Thavorn, K., Catley, C., Hawken, S., Gillespie, U., & Manuel, D.G. (2015). PIP-STOPP Study: Assessing Potentially Inappropriate Prescribing in (PIP) and predicting patient outcomes using a subset of the STOPP criteria in Ontario’s older population: a population-based cohort study using large health administrative databases. BMJ Open, 5(e010146). doi:<http://bmjopen.bmj.com/content/5/11/e010146> 2. Black, C., Thavorn K., Coyle D., Smith G., Bjerre, L.M. (2018). The health system costs of potentially inappropriate prescribing in Ontario, Canada: a protocol for a population-based cohort study. BMJ Open, 8(e021727). doi: <https://bmjopen.bmj.com/content/8/6/e021727> 3. Black, C.D., Thavorn, K., Coyle, D., & Bjerre, L.M. (2020). The health system costs of potentially inappropriate prescribing: a population-based, retrospective cohort study using linked health administrative databases in Ontario, Canada. Pharmacoeconomics Open, 4(1):27-36. doi: [10.1007/s41669-019-0143-2](https://pubmed.ncbi.nlm.nih.gov/31218653/) | | |
| 8 | Indicator name | Older adults who receive a collaborative medication review. | | |
|  | Description | The proportion of attached older adults to a family physician who have been prescribed one or more medications from multiple providers and receive a collaborative medication review in 2019. | | |
|  | Calculation | Proportion, % (Numerator divided by denominator x 100%) | | |
|  | Numerator | The number of older adults (aged ≥65) with a prescription for one or more medications from more than one prescribing physician in 2019. | | |
|  |  | Data set(s) | ODB (MedsCheck Name, DIN): | |
|  |  | Variable(s) | - Annual (93899979), - Follow-up Hospital Discharge (93899981), - Follow-up for Pharmacist Referral (93899982), - Follow-up for MD/RN referral (93899983), - Medication review (93899985), - Follow-up for hospital admission (93899984), - Diabetes Annual Assessment (93899988), - Diabetes Follow-up Assessment (93899989), **or** - Annual at Home (93899987). | |
|  | Denominator | The number of older adults (aged ≥65) attached (formally rostered or virtually) to a particular family physician with one or more prescribing physicians in 2019. | | |
|  |  | Data set(s) | CAPE (main source for rostering information)  OHIP (virtual rostering information)  Patients with more than one prescribing physician ID (PRESC_I) in all ODB records in 2019. | |
|  | Considerations and limitations | 1. There are no specific fee codes for physicians to conduct medication reviews. Therefore, we are limited at only measuring MedChecks activities captured in ODB for pharmacists. Pharmacists working within FHTs who are salaried and will not bill ODB for MedChecks activities. Further, medication reconciliation may be performed by other health care professionals (e.g., home care nurses patient discharge from hospital). The numerator will likely be underreported. 2. We do not know the extent of the medication reconciliation activities (e.g., whether all four activities outlined by the Institute for Safe Medication Practices were conducted). | | |
|  | References | Ontario Ministry of Health. (2024). Professional pharmacy services. Available from: <https://www.ontario.ca/page/professional-pharmacy-services> | | |
| 9 | Indicator name | Older adults living with CHF who are prescribed ACE inhibitors, ARBs, beta-blockers, or SGLT2 inhibitors. | | |
|  | Description | The proportion of older adults living with CHF who were prescribed ACE inhibitors, ARBs, beta-blockers, or SGLT2 inhibitors in 2019. | | |
|  | Calculation | Proportion, % (Numerator divided by denominator x 100%) | | |
|  | Numerator | The number of older adults (aged ≥65) whose family physician billed for administering influenza and pneumococcal immunizations in 2019. | | |
|  |  | Data set(s) | ODB DINs: | |
|  |  | Variable(s) | - ACE inhibitors: 02273918, 02290332, 02290340, 00893595, 00893609, 00893617, 00893625, 01942964, 01942972, 01942980, 01942999, 02266350, 02266369, 02266377, 02283778, 02283786, 02283794, 02291134, 02291142, 02291150, 01911473, 01911481, 02444771, 02444798, 02459450, 02459469, 02474786, 02474794, 02474808, 02474816, 00670901, 00670928, 00708879, 02019884, 02019892, 02019906, 02020025, 02233005, 02233006, 02233007, 02291878, 02291886, 02291894, 02291908, 02299933, 02299941, 02299968, 02299976, 02300036, 02300044, 02300052, 02300060, 02300087, 02300095, 02300109, 02300117, 02300125, 02300133, 02300141, 02300680, 02352230, 02352249, 02352257, 02352265, 02331004, 02247802, 02247803, 02266008, 02266016, 02294524, 02294532, 02301768, 02045737, 02103729, 02301776, 02302136, 02302144, 02302365, 02302373, 02408767, 02408783, 00839396, 00839418, 02049333, 02049376, 02049384, 02217481, 02217503, 02217511, 02271443, 02271451, 02271478, 02285061, 02285088, 02285096, 02285118, 02285126, 02285134, 02289199, 02289202, 02289229, 02292211, 02292238, 02294230, 02294249, 02294257, 02361531, 02361558, 02361566, 02394472, 02394480, 02394499, 09853685, 09853960, 09854010, 09857272, 09857286, 09857287, 02123274, 02123282, 02246624, 02289261, 02289288, 02289296, 02459817, 02459825, 02459833, 02464985, 02464993, 02465000, 02470225, 02470233, 02470241, 02470675, 02470683, 02470691, 02474824, 02474832, 02474840, 02476762, 02476770, 02476789, 02477009, 02477017, 02477025, 02248499, 02248500, 02248501, 02248502, 01947664, 01947672, 01947680, 01947699, 02290995, 02291002, 02291010, 02340550, 02340569, 02340577, 02340585, 02221829, 02221837, 02221845, 02221853, 02247917, 02247918, 02247919, 02247945, 02247946, 02247947, 02251515, 02251531, 02251574, 02251582, 02287706, 02287927, 02287935, 02287943, 02295369, 02295482, 02295490, 02295504, 02295512, 02310503, 02310511, 02310538, 02310546, 02331101, 02331128, 02331136, 02331144, 02374846, 02374854, 02374862, 02387387, 02387395, 02387409, 02387417, 02469057, 02469065, 02469073, 02469081, 02420457, 02420465, 02420473, 02420481, 02421305, 02421313, 02421321, 02438887, 02231459, 02231460, 02239267, 02325748, 02325756, 02325764, 02357763, 02357771, 02357798, 02415437, 02415445, 02415453, 02471876, 02471884, **or** 02471892, - ARBs: 02239090, 02239091, 02239092, 02311658, 02326957, 02326965, 02326973, 02365340, 02365359, 02365367, 02366312, 02366320, 02366339, 02376520, 02376539, 02376547, 02376555, 02379120, 02379139, 02379147, 02379155, 02379260, 02379279, 02379287, 02379295, 02380684, 02380692, 02380706, 02380714, 02386496, 02386518, 02386526, 02386534, 02391171, 02391198, 02391201, 02391228, 02399105, 02445786, 02445794, 02445808, 02445816, 02476916, 02476924, 02417340, 02240432, 02243942, 02253631, 02244344, 02318709, 02393263, 02393271, 02393557, 02393565, 02419114, 02419122, 02420023, 02420031, 02456389, 02456397, 02237923, 02237924, 02237925, 02316390, 02316404, 02316412, 02317060, 02317079, 02317087, 02328070, 02328089, 02328100, 02328461, 02328488, 02328496, 02347296, 02347318, 02386968, 02386976, 02386984, 02406810, 02406829, 02406837, 02418193, 02418207, 02418215, 02422980, 02422999, 02423006, 02406098, 02406101, 02406128, 02445980, 02182815, 02182874, 02182882, 02309750, 02309769, 02309777, 02313332, 02313340, 02313359, 02353504, 02353512, 02354829, 02354837, 02354845, 02357968, 02357976, 02368277, 02368285, 02368293, 02379058, 02380838, 02398834, 02398842, 02398850, 02403323, 02403331, 02403358, 02404478, 02404486, 02405733, 02405741, 02405768, 02422484, 02424967, 02424975, 02424983, 02426595, 02442191, 02442205, 02443414, 02443422, 02443864, 02443872, 02453452, 02453460, 02461307, 02461315, 02461641, 02461668, 02318660, 02318679, 02240769, 02240770, 02320177, 02320185, 02375958, 02375966, 02376717, 02376725, 02393247, 02393255, 02432897, 02432900, 02453568, 02453576, 02407485, 02407493, 02420082, 02420090, 02434164, 02244781, 02244782, 02289504, 02313006, 02337495, 02337509, 02337517, 02356651, 02356678, 02356686, 02356759, 02356767, 02356775, 02363100, 02363119, 02371529, 02371537, 02371545, 02383535, 02414228, 02414236, **or** 02414244, - Beta blockers: 01926543, 01926551, 02147602, 02147610, 02147629, 02204517, 02204525, 02204533, 02237721, 02237722, 02237723, 02466465, 02466473, 00773689, 00773697, 01912054, 01912062, 02039532, 02039540, 02146894, 02147432, 02171791, 02171805, 02237600, 02237601, 02255545, 02255553, 02267985, 02267993, 02367564, 02367572, 02368021, 02368048, 02368641, 02371987, 02371995, 02465612, 02465620, 02247439, 02247440, 02256134, 02256177, 02267470, 02267489, 02245914, 02245915, 02245916, 02245917, 02247933, 02247934, 02247935, 02247936, 02248752, 02248753, 02248755, 02252309, 02252317, 02252325, 02252333, 02268027, 02268035, 02268043, 02268051, 02364913, 02364921, 02364948, 02364956, 02368897, 02368900, 02368919, 02368927, 02418495, 02418509, 02418517, 02418525, 02106272, 02106280, 02243538, 02243539, 02489406, 02489414, 00618632, 00618640, 00749354, 00751170, 00842648, 00842656, 02230803, 02230804, 02354187, 02354195, 02356821, 02356848, 00648035, 00648043, 02303396, 02303418, 02350394, 02350408, 02285169, 02285177, 00534560, 00658855, 00782467, 00782475, 00782505, 00417270, 00443174, 00755877, 00755885, 00755893, 00869007, 00869015, 00869023, 02042258, 02042266, 02042274, 02457857, 00496480, 00496499, 00496502, 00740675, 02084236, 02167794, 02210428, 02231182, 0223832, 02238327, 02270633, 02368617, 02368625, 00755842, 00755850, 00755869, 00451207, 00755826, 00755834, 02083345, 02083353, 02166712, 02166720, 02171880, 02171899, 02242275, 02242276, **or** 02290812, - SGLT2 inhibitors: 02425483, 02425491, 02435462, 02435470, 02449935, 02449943, 02443937, 02443945, 02456575, 02456583, 02456591, 02456605, 02456613, **or** 02456621 | |
|  |  |  | Note: OHIP/ODB SERVDATE for activities occurred after dementia diagnosis date. | |
|  | Denominator | The number of older adults (aged ≥65) living with CHF attached (formally rostered or virtually) to a particular family physician in 2019. | | |
|  |  | Data set(s) | CAPE (main source for rostering information)  OHIP (virtual rostering information)  CHF (ICES-derived cohort) | |
|  | Considerations and limitations | 1. There is no concern about construct validity as the CHF derived cohort is not informed by ODB claims. 2. The family physician may not have prescribed the relevant medications (e.g., prescribed by cardiologist or internal medicine consult); the indicator may not accurately reflect their clinical practice. 3. There are instances where it is not appropriate to prescribe these medications for clinical reasons. However, in the absence of patient-level clinical data, we cannot exclude CHF patients who were deemed ineligible to receive these medications. Further, these prescriptions may only be warranted based on clinical symptoms (e.g., reduced ejection fraction), which cannot be discerned from administrative data. | | |
|  | References | This DIN list was based on an extensive search in the ODB at ICES (using the %dinexplore macro) for all active and relevant drugs, and reviewed for accuracy by a physician. | | |
| 10 | Indicator name | Older adults living with dementia who are prescribed antipsychotics. | | |
|  | Description | The proportion of older adults living with dementia who are prescribed antipsychotics in 2019. | | |
|  | Calculation | Proportion, % (Numerator divided by denominator x 100%) | | |
|  | Numerator | The number of older adults (aged ≥65) with ODB orders for antipsychotics in 2019. | | |
|  |  | Data set(s) | ODB DINs: | |
|  |  | Variable(s) | - 09900065, 02420864, 02420872, 02322374, 02322382, 02322390, 02322404, 02322412, 02322455, 02460025, 02460033, 02460041, 02460068, 02460076, 02460084, 02464144, 02464152, 02464160, 02464179, 02464187, 02464195, 02466635, 02466643, 02466651, 02466678, 02466686, 02466694, 02471086, 02471094, 02471108, 02471116, 02471124, 02471132, 02473658, 02473666, 02473674, 02473682, 02473690, 02473704, 02374811, 02374803, 00232807, 00232823, 00232831, 02156040, 02156032, 02156008, 02156016, 00755575, 00405345, 00405361, 00410632, 00363650, 00363669, 00363677, 00363685, 00713449, 00768820, 09853758, 00808652, 02230838, 02230839, 02230840, 02230837, 01927698, 02238403, 02238404, 02238405, 02238406, 02229250, 02229269, 02229277, 02229285, 02238850, 02276712, 02276720, 02276739, 02276747, 02276755, 02281791, 02281805, 02281813, 02281821, 02281848, 02303116, 02303159, 02303167, 02303175, 02303183, 02310341, 02310368, 02310376, 02310384, 02310392, 02325659, 02325667, 02325675, 02325683, 02325691, 02337878, 02337886, 02337894, 02337908, 02337916, 02372819, 02372827, 02372835, 02372843, 02372851, 02403072, 02403099, 02403102, 02410141, 02410184, 02243086, 02243087, 02243088, 02303191, 02303205, 02303213, 02321343, 02321351, 02321378, 02327562, 02327570, 02327589, 02327775, 02327783, 02327791, 02360616, 02360624, 02360632, 02389088, 02389096, 02389118, 02406624, 02406632, 02406640, 02414090, 02414104, 02417243, 02417251, 02417278, 02417286, 02417294, 02421232, 02421240, 02421259, 02421267, 02421275, 02414112, 02436965, 02436973, 02436981, 02448734, 02448742, 02448726, 02300273, 02300281, 02300303, 02354217, 02354225, 02354233, 02354241, 02455943, 02455986, 02455994, 02456001, 01926772, 01926780, 01926756, 00335096, 00335118, 00335126, 00335134, 00313815, 02245432, 02245433, 00789720, 02457229, 02457237, 02457245, 02457253, 02457261, 02438046, 02447193, 02395479, 02407671, 02407698, 02407701, 02407728, 02407736, 02236951, 02236952, 02236953, 02244107, 02284235, 02284243, 02284278, 02284286, 02296551, 02296578, 02296594, 02296608, 02313901, 02313928, 02313936, 02313944, 02313995, 02314002, 02314010, 02314029, 02316080, 02316099, 02316110, 02316129, 02317893, 02317907, 02317923, 02317931, 02330415, 02330423, 02330458, 02330466, 02353164, 02353199, 02387794, 02387808, 02387824, 02387832, 02390205, 02390213, 02390248, 02390256, 02397099, 02397102, 02397110, 02397129, 02399822, 02399830, 02399849, 02399857, 02438003, 02438011, 02438054, 02300184, 02300192, 02300206, 02300214, 02321513, 02395444, 02395452, 02395460, 02395487, 02434024, 02439158, 02439166, 02439182, 02439190, 02255707, 02255723, 02255758, 02236950, 02279266, 02280396, 02025280, 02025299, 02025302, 02025310, 02240551, 02240552, 02252007, 02252015, 02252023, 02252031, 02252058, 02252066, 02264188, 02264196, 02264218, 02264226, 02264234, 02279800, 02279819, 02279827, 02279835, 02282119, 02282127, 02282135, 02282143, 02282151, 02282178, 02282585, 02282593, 02282607, 02282615, 02282623, 02282631, 02282690, 02303655, 02303663, 02328305, 02328313, 02328321, 02328348, 02328364, 02328372, 02356880, 02356899, 02356902, 02356910, 02356929, 02356937, 02359529, 02359537, 02359545, 02359553, 02359561, 02359588, 02359790, 02359804, 02359812, 02359820, 02359839, 02359847, 02371766, 02371774, 02371782, 02371790, 02371804, 02371812, 02247705, 02291789, 02291797, 02370697, 02413493, 02413507, 02413515, 02413523, 02413485, 02454319, 00312746, 00312754, 00326836, 00345539, 02298597, 02298600, 02298619, 02298627, 02449544, 02449552, 02449560, 02449579, 02230406, 02230402, **or** 02230403 | |
|  |  |  | Note: ODB SERVDATE for activities occurred after dementia diagnosis date. | |
|  | Denominator | The number of older adults (aged ≥65) living with dementia attached (formally rostered or virtually) to a particular family physician in 2019. | | |
|  |  | Data set(s) | CAPE (main source for rostering information)  OHIP (virtual rostering information)  DEMENTIA (ICES-derived cohort) | |
|  | Considerations and limitations | 1. This indicator does not consider clinical or behavioural symptoms of patients that may warrant antipsychotic prescriptions (e.g., if patient is aggressive towards family caregivers). | | |
|  | References | Mast, G., Fernandes, K., Tadrous, M., Martins, D., Herrmann, N., & Gomes, T. (2016). Persistence of antipsychotic treatment in elderly dementia patients: a retrospective, population-based cohort study. Drugs-Real World Outcomes, 3, 175-182.  Alzheimer Society. (2024). The risk of using antipsychotic medications to treat dementia. Available: <https://alzheimer.ca/en/about-dementia/how-can-i-treat-dementia/risk-using-antipsychotic-medications-treat-dementia> | | |
| 11 | Indicator name | Older adults living with dementia who were reported to Ministry of Transportation. | | |
|  | Description | The proportion of older adults living with dementia who were reported to Ministry of Transportation in 2019. | | |
|  | Calculation | Proportion, % (Numerator divided by denominator x 100%) | | |
|  | Numerator | The number of older adults (aged ≥65) whose family physician billed for reporting a medical condition to the Ontario Ministry of Transportation. | | |
|  |  | Data set(s) | OHIP fee codes: | |
|  |  | Variable(s) | - K035 (Mandatory reporting of medical condition to the Ontario Ministry of Transportation)   Note: OHIP SERVDATE for activities occurred after dementia diagnosis date. | |
|  | Denominator | The number of older adults (aged ≥65) living with dementia attached (formally rostered or virtually) to a particular family physician in 2019. | | |
|  |  | Data set(s) | CAPE (main source for rostering information)  OHIP (virtual rostering information)  DEMENTIA (ICES-derived cohort) | |
|  | Considerations and limitations | 1. Without knowledge of patients’ clinical or behavioural symptoms, we do not know whether instances of reporting to the Ministry of Transportation were warranted and appropriate (e.g., if the patient was assessed as unsafe to drive by their family physician). 2. The family physician may not have completed the reporting (e.g., may have be reported by neurologist); the indicator may not accurately reflect their clinical practice. | | |
|  | References | Baycrest Centre for Geriatric Care and Sunnybrook Health Sciences Centre. (2022). Driving & Dementia Roadmap. Available: <https://www.drivinganddementia.ca/> | | |

OHIP=Ontario Health Insurance Plan; ODB=Ontario Drug Benefit; DIN=Drug Identification Number; CAPE=Client Agency Program Enrolment; FHT=Family Health Team; DEMENTIA=ICES-derived cohort of persons living with dementia; COPD=ICES-derived cohort of persons living with chronic obstructive pulmonary disease; ACE=angiotensin-converting enzyme; ARBs=angiotensin 2 receptor blockers; SGLT2=sodium-glucose co-transporter-2; CHF=ICES-derived cohort of persons living with congestive heart failure.

**S4 Appendix. Summary of missing data**

|  | | **Sample size** (Percentage of sample included in indicator computation) | |
| --- | --- | --- | --- |
|  |  | **FPs with a focused practice or certification in COE**  (n=232) | **FPs without a focused practice or certification in COE**  (n=928) |
|  | **Medical Conditions** | | |
| **Indicator 1:** Proportion of attached patients aged ≥65 who received the influenza vaccine, % | | 168 (72.41%) | 672 (72.41%) |
| **Indicator 2:** Proportion of attached patients aged ≥65 living with COPD who received influenza and pneumococcal immunizations, % | | 126 (54.31%) | 504 (54.31%) |
| **Indicator 3^^^:** Proportion of attached patients aged ≥65 living with dementia who received tests aligned with the most current Canadian Consensus on Dementia, % | | 114 (49.14%) | 456 (49.14%) |
| **Indicator 4^^^:** Proportion of attached patients aged ≥65 living with dementia who received dementia care management, % | | 114 (49.14%) | 456 (49.14%) |
|  | **Appropriate Prescribing** | | |
| **Indicator 5:** Proportion of attached patients aged ≥65 who are prescribed one or more benzodiazepines, % | | 168 (72.41%) | 672 (72.41%) |
| **Indicator 6:** Proportion of attached patients aged ≥65 who are prescribed one or more medications with strong anticholinergic effects, % | | 168 (72.41%) | 672 (72.41%) |
| **Indicator 7:** Proportion of attached patients aged ≥65 who are prescribed one or more potentially inappropriate medications (e.g., from Beers list, START/STOPP criteria), % | | 168 (72.41%) | 672 (72.41%) |
| **Indicator 8:** Proportion of attached patients aged ≥65 with more than one prescribing physician who received a collaborative medication review, % | | 159 (68.53%) | 636 (68.53%) |
| **Indicator 9:** Proportion of attached patients aged ≥65 living with CHF who were prescribed ACE inhibitors, ARBs, beta-blockers, or SGLT2 inhibitors, % | | 107 (46.12%) | 428 (46.12%) |
| **Indicator 10^^^:** Proportion of attached patients aged ≥65 living with dementia who are prescribed antipsychotics, % | | 114 (49.14%) | 456 (49.14%) |
|  | **Driving Issues** | | |
| **Indicator 11^^^:** Proportion of attached patients aged ≥65 living with dementia whose medical condition was reported to the Ministry of Transportation, % | | 114 (49.14%) | 456 (49.14%) |
| FP=family physician; COE=Care of the Elderly; COPD=chronic obstructive pulmonary disease; CHF=congestive heart failure; ACE=Angiotensin-converting-enzyme; ARBs= Angiotensin receptor blockers; SGLT2=Sodium-glucose cotransporter-2  ^^^ Indicator also relates to the Cognitive Impairment COE Priority Topic | | | |

**S5 Appendix. Sensitivity analysis**

|  | **FPs with a focused practice or certification in COE**  Mean (SD) | | | **No focused practice or certification in COE**  Mean (SD) | **P value** |
| --- | --- | --- | --- | --- | --- |
|  | Certification in COE only | Both a focused practice and certification in COE | Focused practice in COE only |  |  |
| **Medical Conditions** | | | | | |
| **Indicator 1:** Proportion of attached patients aged ≥65 who received the influenza vaccine, % | 25.93 (23.88) | 22.86 (28.05) | 36.63 (25.83) | 30.53 (24.60) | 0.0258 * |
| **Indicator 2:** Proportion of attached patients aged ≥65 living with COPD who received influenza and pneumococcal immunizations, % | 23.35 (22.28) | 29.12 (29.80) | 41.13 (24.22) | 32.24 (25.81) | 0.0041 * |
| **Indicator 3^^^:** Proportion of attached patients aged ≥65 living with dementia who received tests aligned with the most current Canadian Consensus on Dementia, % | 4.08 (6.76) | 1.86 (3.73) | 5.64 (8.69) | 2.60 (6.52) | 0.0123 * |
| **Indicator 4^^^:** Proportion of attached patients aged ≥65 living with dementia who received dementia care management, % | 58.90 (30.14) | 73.33 (25.53) | 80.20 (19.72) | 68.75 (27.58) | 0.0016 * |
| **Appropriate Prescribing** | | | | | |
| **Indicator 5:** Proportion of attached patients aged ≥65 who are prescribed one or more benzodiazepines, % | 9.25 (7.01) | 12.74 (12.56) | 9.24 (12.37) | 8.95 (9.69) | 0.2796 |
| **Indicator 6:** Proportion of attached patients aged ≥65 who are prescribed one or more medications with strong anticholinergic effects, % | 0.13 (0.38) | 0.01 (0.05) | 0.05 (0.14) | 0.11 (0.55) | 0.5298 |
| **Indicator 7:** Proportion of attached patients aged ≥65 who are prescribed one or more potentially inappropriate medications (e.g., from Beers list, START/STOPP criteria), % | 40.19 (22.36) | 45.85 (26.94) | 34.81 (18.16) | 30.45 (19.73) | <.0001 * |
| **Indicator 8:** Proportion of attached patients aged ≥65 with more than one prescribing physician who received a collaborative medication review, % | 0.09 (0.26) | 0.01 (0.07) | 0.05 (0.19) | 0.09 (0.51) | 0.7901 |
| **Indicator 9:** Proportion of attached patients aged ≥65 living with CHF who were prescribed ACE inhibitors, ARBs, beta-blockers, or SGLT2 inhibitors, % | 56.65 (23.73) | 52.42 (26.93) | 64.78 (21.27) | 57.15 (25.22) | 0.2262 |
| **Indicator 10^^^:** Proportion of attached patients aged ≥65 living with dementia who are prescribed antipsychotics, % | 20.63 (15.39) | 26.06 (20.56) | 17.69 (20.85) | 15.17 (15.15) | 0.006 * |
| **Driving Issues** | | | | | |
| **Indicator 11^^^:** Proportion of attached patients aged ≥65 living with dementia whose medical condition was reported to the Ministry of Transportation, % | 1.18 (4.89) | 0.72 (1.20) | 2.51 (5.68) | 0.98 (2.99) | 0.0313 * |
| FP=family physician; COE=Care of the Elderly; COPD=chronic obstructive pulmonary disease; CHF=congestive heart failure; ACE=Angiotensin-converting-enzyme; ARBs= Angiotensin receptor blockers; SGLT2=Sodium-glucose cotransporter-2  *Significant at the level of 0.05  ^^^ Indicator also relates to the Cognitive Impairment ‘Care of the Elderly’ Priority Topic | | | | | |

1. Approach to measuring long-term care practice was described in: Correia, R. H., Dash, D., Poss, J. W., Moser, A., Katz, P. R., & Costa, A. P. (2022). Physician Practice in Ontario Nursing Homes: Defining Physician Commitment. *Journal of the American Medical Directors Association*, *23*(12), 1942–1947.e2. https://doi.org/10.1016/j.jamda.2022.04.011 [↑](#footnote-ref-1)
